# High levels of cerebrospinal fluid chemokines point to the presence of neuroinflammation in peripheral neuropathic pain: a cross-sectional study of 2 cohorts of patients compared with healthy controls

**DOI:** 10.1097/j.pain.0000000000001061

**Published:** 2017-09-18

**Authors:** Emmanuel Bäckryd, Anne-Li Lind, Måns Thulin, Anders Larsson, Björn Gerdle, Torsten Gordh

**Affiliations:** a Pain and Rehabilitation Centre, and Department of Medical and Health Sciences, Linköping University, Linköping, Sweden; b Department of Surgical Sciences, Anesthesiology and Intensive Care, and Uppsala Berzelii Technology Center for Neurodiagnostics, Uppsala University, Uppsala, Sweden; Departments of c Statistics and; d Medical Sciences, Uppsala University, Uppsala, Sweden

**Keywords:** Biomarker, Cerebrospinal fluid, Chemokines, Cytokines, Human, Inflammation, Neuroinflammation, Neuropathic pain, Protein profile, Proximity extension assay

## Abstract

Animal models suggest that chemokines are important mediators in the pathophysiology of neuropathic pain. Indeed, these substances have been called “gliotransmitters,” a term that illustrates the close interplay between glial cells and neurons in the context of neuroinflammation and pain. However, evidence in humans is scarce. The aim of the study was to determine a comprehensive cerebrospinal fluid (CSF) inflammatory profile of patients with neuropathic pain. Our hypothesis was that we would thereby find indications of a postulated on-going process of central neuroinflammation. Samples of CSF were collected from 2 cohorts of patients with neuropathic pain (n = 11 and n = 16, respectively) and healthy control subjects (n = 11). The samples were analyzed with a multiplex proximity extension assay in which 92 inflammation-related proteins were measured simultaneously (Proseek Multiplex Inflammation I; Olink Bioscience, Uppsala, Sweden). Univariate testing with control of false discovery rate, as well as orthogonal partial least squares discriminant analysis, were used for statistical analyses. Levels of chemokines CXCL6, CXCL10, CCL8, CCL11, CCL23 in CSF, as well as protein LAPTGF-beta-1, were significantly higher in both neuropathic pain cohorts compared with healthy controls, pointing to neuroinflammation in patients. These 6 proteins were also major results in a recent similar study in patients with fibromyalgia. The findings need to be confirmed in larger cohorts, and the question of causality remains to be settled. Because it has been suggested that prevalent comorbidities to chronic pain (eg, depression, anxiety, poor sleep, and tiredness) also are associated with neuroinflammation, it will be important to determine whether neuroinflammation is a common mediator.

## Abstract 2

According to animal models, neuroinflammation is a major feature of neuropathic pain. The present findings confirm that this hypothesis is of relevance to humans.

## 1. Introduction

Neuropathic pain (NeuP) is defined as pain caused by a lesion or disease of the somatosensory nervous system.^33^ The prevalence of chronic pain with neuropathic characteristics in the general population has been estimated to be up to 7%.^8^ Available analgesics often have limited effects or lead to troublesome side effects.^5,27^ Current evidence indicates that at least 6 patients have to be treated with a first-line drug (eg, serotonin–norepinephrine reuptake inhibitors or gabapentinoids) in order for 1 patient to obtain clinically significant pain relief.^27^

When trying to better understand what causes and maintains NeuP, it is important to move beyond mere etiology and study the pathophysiological mechanisms involved, for instance by in-depths somatosensory phenotyping.^70^ Another way forward is to study the biochemical profile of NeuP patients using 'omics methodology.^2,10,64,79^ The cerebrospinal fluid (CSF) seems to be a sensible biofluid to investigate in pain conditions, as it can reasonably be hypothesized to mirror central nervous system pathology. For instance, CSF levels of classical neuropeptides, like substance P and beta-endorphin (and other endogenous opioids), have historically been studied in many different pain states.^1,4,11,65,73,74^

Much of our knowledge concerning the pathophysiological mechanisms of NeuP has been gained from animal experiments. It has become increasingly clear that immunocompetent glial cells, such as microglia and astrocytes, are key contributors to the pathophysiology of chronic NeuP.^6,14,24,31,32,51,60,76,78^ Hence, central neuroimmune and neuroinflammatory mechanisms are nowadays considered to be very important in the pathophysiology of NeuP. However, it is important to stress that this has mainly been shown in preclinical models of chronic pain and that evidence in humans is less clear.^14,32,60^ Indeed, glial cells (at least astrocytes) from mice and monkeys are quite different from their human counterparts.^71^ Translating evidence from animals to humans is far from trivial,^50^ but a series of recent studies using a comprehensive panel of 92 inflammation-related proteins indicate the presence of low-grade systemic inflammation and neuroinflammation in chronic widespread pain conditions^13,30^ and of systemic inflammation in chronic lumbar radicular pain.^55^

Cytokines and chemokines are thought to be important mediators in the pathophysiology of NeuP, at least in preclinical models.^18,42,60^ Indeed, chemokines and other pronociceptive mediators in the spinal cord have been called “gliotransmitters,”^60^ a term that illustrates the close interplay between glial cells and neurons in the context of neuroinflammation and chronic pain.

The aim of the present study was to use a multiplex panel allowing the measuring of 92 inflammation-related proteins in a single run^55^ and apply it to the CSF of patients with peripheral NeuP and healthy control subjects. Our hypothesis was that we would be able to determine a CSF inflammatory profile of NeuP patients and that we would be able to mirror a postulated on-going process of central neuroinflammation.

## 2. Methods

First, we compared NeuP patients (called cohort 1a) and healthy control subjects (cohort 1b) recruited at the same center (Linköping, Sweden). Then, to test the reproducibility of our results, an additional cohort of patients (called cohort 2) with a similar pain condition but belonging to another center (Uppsala, Sweden) were compared with cohort 1b.

### 2.1. Procedures

For every subject in this study, intrathecal access was obtained by lumbar puncture and a 10-mL sample of CSF was taken. Details of the CSF sampling procedure have been published earlier and are not repeated here.^10,46^

### 2.2. Subjects

Following the criteria of Treede et al.,^72^ all patients in cohort 1a had a least probable NeuP and patients in cohort 2 had definite NeuP. All cohort 1a patients included in this study were also participating in a clinical trial of intrathecal bolus injections of the analgesic ziconotide, CSF being sampled *before* the injection of ziconotide.^12^ Inclusion criteria were as follows: (1) patient, at least 18 years of age, experiencing chronic (≥6 months) NeuP due to trauma or surgery, who had failed on conventional pharmacological treatment; (2) average visual analogue scale pain intensity in the previous week of ≥40 mm; (3) patient capable of judgment, that is, able to understand information regarding the drug, the mode of administration, and evaluation of efficacy and side effects; (4) signed informed consent. Exclusion criteria and other registered characteristics have been extensively described elsewhere.^10^ All patients were or had been candidates for spinal cord stimulation (SCS).

Age-matched and sex-matched healthy control subjects (cohort 1b) were recruited by local advertisements at the Faculty of Health Sciences, Linköping University, Sweden.^10^

Cohort 2 consisted of patients being treated with SCS. The SCS treatment was turned off during 2 days, whereupon a lumbar puncture was performed. Details about cohort 2 have been published elsewhere.^46^

### 2.3. Proximity extension assay

We used a multiplex proximity extension assay (PEA) in which 92 proteins (see supplemental digital content 1, available online at http://links.lww.com/PAIN/A482) are simultaneously analyzed.^3,47,55^ The multiplex PEA was conducted using Proseek Multiplex Inflammation I (Olink Bioscience, Uppsala, Sweden) according to the manufacturer's instructions. Briefly, 1-μL sample was mixed with 3-μL incubation mixture containing 94 probe pairs (each pair consisting of 2 target-specific antibodies equipped with unique barcoded DNA oligonucleotides). The mixture was incubated at 8°C overnight. Then, 96-μL extension mixture containing PEA enzyme and polymerase chain reaction reagents was added, incubated for 5 minutes at room temperature before the plate was transferred to a thermal cycler for an extension reaction followed by 17 cycles of DNA amplification. A 96.96 Dynamic Array IFC (Fluidigm, South San Francisco, CA) was prepared and primed according to the manufacturer's instructions. In a new plate, 2.8 μL of sample mixture was mixed with 7.2 μL of detection mixture from which 5 μL was loaded into the right side of the primed 96.96 Dynamic Array IFC. Five microliters of the primer pairs, unique for each assay, was loaded into the left side of the 96.96 Dynamic Array IFC, and the protein expression program was run in Fluidigm Biomark reader (Fluidigm Corporation) according to the instructions of Proseek Multiplex. Data are expressed as normalized protein expression (NPX). Values of NPX are acquired by normalizing cq values against extension control, as well as interplate control and a correction factor. They are on log2 scale. A high NPX value corresponds to a high protein concentration and can be linearized using the formula 2^NPX^. Also, NPX can be used for statistical multivariate analysis and express relative quantification between samples but is not an absolute quantification. Data showing the correlation between the present PEA method and an electrochemiluminescence immunoassay (Meso Scale Discovery MULTI-ARRAY technology) for plasma CXCL1 and CXCL10 is shown in supplemental digital content 1 (available online at http://links.lww.com/PAIN/A482), where a link to the extensive background information on the method available online is also provided.

### 2.4. Statistics

When comparing the demographics of patients and healthy control subjects, data are shown as median (range), and the Mann–Whitney *U* test or Fisher exact test was used as appropriate for inferential statistics (version 23, IBM SPSS statistics, IBM Corporation, Armonk, NY).

Proteins with more than 20% of values below the limit of detection were excluded from further analysis.^55^ For each protein, we tested whether there was a difference in expression levels between the 2 groups using a 2-sided Mann–Whitney *U* test. Performing such a large number of tests increases the risk of false discoveries. Therefore, we adjusted the *P* values for multiplicity using the false discovery rate (FDR) approach.^7^

We also used multivariate data analysis by projection with the SIMCA software version 13 (Umetrics AB, Umeå, Sweden).^10,25,79^ The statistical workflow has been extensively described elsewhere,^10,59^ and it is consistent with the recommendations issued by Wheelock and Wheelock.^79^ Multivariate data analysis by projection analyzes all the variables together at the same time, taking the correlation structure of the data set into consideration, thereby favoring structure and information over “noise” and false-positive findings.^25^ Briefly, data were first overviewed by principal components analysis (PCA) (which conceptually can be viewed as a multivariate correlation analysis). However, PCA was used here for the identification of outliers and deviant subgroups in the data. Then, orthogonal partial least squares discriminant analysis (OPLS-DA) (ie, a regression technique) was used to identify the proteins (ie, *X* variables) most responsible for class discrimination (dichotomous *Y* variable). The statistical significance of the regression is expressed using the *P* value of the cross-validated analysis of variance (CV-ANOVA). The strength of class separation can be visualized in a plot showing how each individual subject relates to the 2 first latent variables of the model (score plot). The relative importance of each protein (*X* variable) for class discrimination is given by the variable influence on projection (VIP), VIP >1 indicating that the variable has an above-average influence on class discrimination (*Y* variable).^25^ In this study, a VIP cutoff of 1.3 was chosen for reporting interesting class-discriminating proteins.

### 2.5. Ethics

The protocol of healthy control subjects was approved by the Regional Ethics Committee in Linköping, Sweden (Dnr M136-06 and Dnr 2012/94-32). The clinical trial, from which patient data from Linköping were derived, was conjointly approved by the Swedish Medical Products Agency (EudraCT 2010-018,920-21) and by the Regional Ethics Committee in Linköping (Dnr 2011/48-31). The study was also approved by the Regional Ethics Committee in Uppsala (01-367). The study was conducted according to the Declaration of Helsinki.

## 3. Results

### 3.1. Patients with neuropathic pain (cohort 1a) versus healthy control subjects (cohort 1b)

Patients with NeuP (cohort 1a, n = 11) and healthy control subjects (cohort 1b, n = 11) did not differ significantly concerning age (57 [39-65] years vs 54 [44-57] years, respectively; *P* = 0.088) and sex (55% vs 64% women, respectively; *P* = 1.0). For detailed individual characteristics of patients of cohort 1a, see Table 1.

**Table 1:**
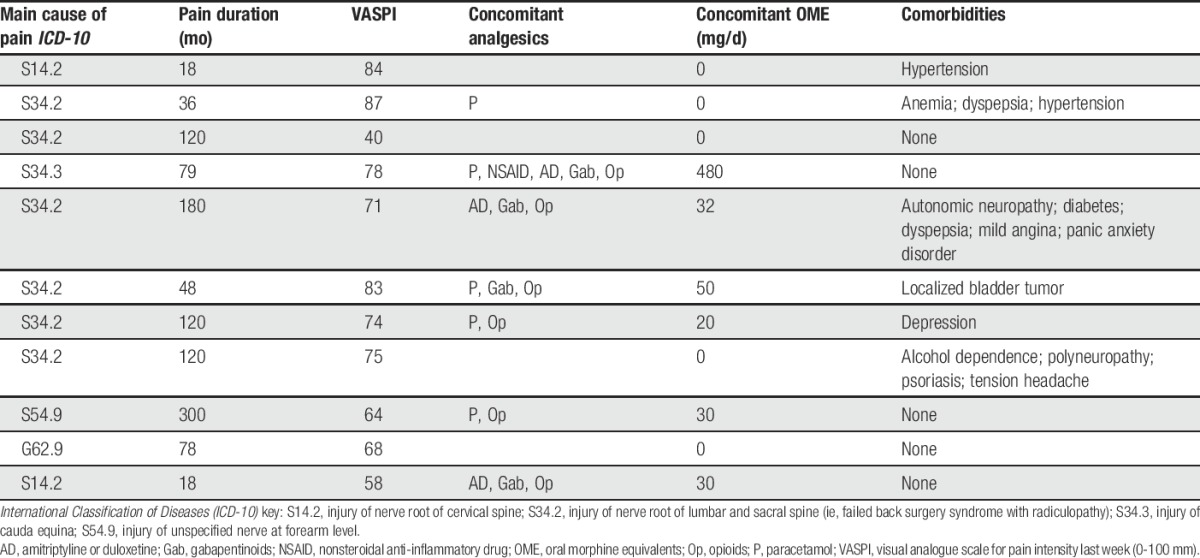
Characteristics of patients with neuropathic pain for cohort 1a (n = 11).

**Figure 1.**
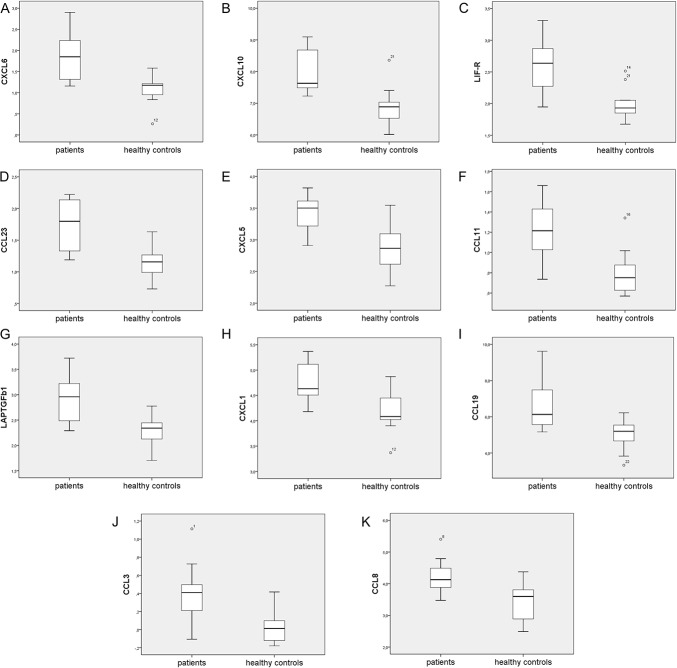
Expression of the 11 most group-discriminating inflammation-related proteins in the cerebrospinal fluid of neuropathic pain patients (cohort 1a) versus healthy control subjects (cohort 1b). The protein levels (y-axis) are expressed as normalized protein expression, as described in the proximity extension assay subsection. Median values are represented by horizontal lines and the interquartile ranges by boxes. The ends of the whiskers depict the lowest and highest datum within 1.5 interquartile range of the lower or upper quartile, respectively. Points represent outliers.

Multiple univariate tests with control of the FDR: 42 of 92 inflammation-related proteins had more than 20% of values below limit of detection and were therefore excluded from analysis. Hence, the following results pertain to the levels of 50 inflammation-related proteins. At an FDR of 10%, the following inflammation-related proteins were significantly associated with NeuP when comparing cohorts 1a and 1b: CXCL1, CXCL5, CXCL6, CXCL10, CCL3, CCL8, CCL11, CCL19, CCL23, LAPTGF-beta-1, and LIF-R (Table 2 and Fig. 1). Nine of these 11 proteins were chemokines.

**Table 2:**
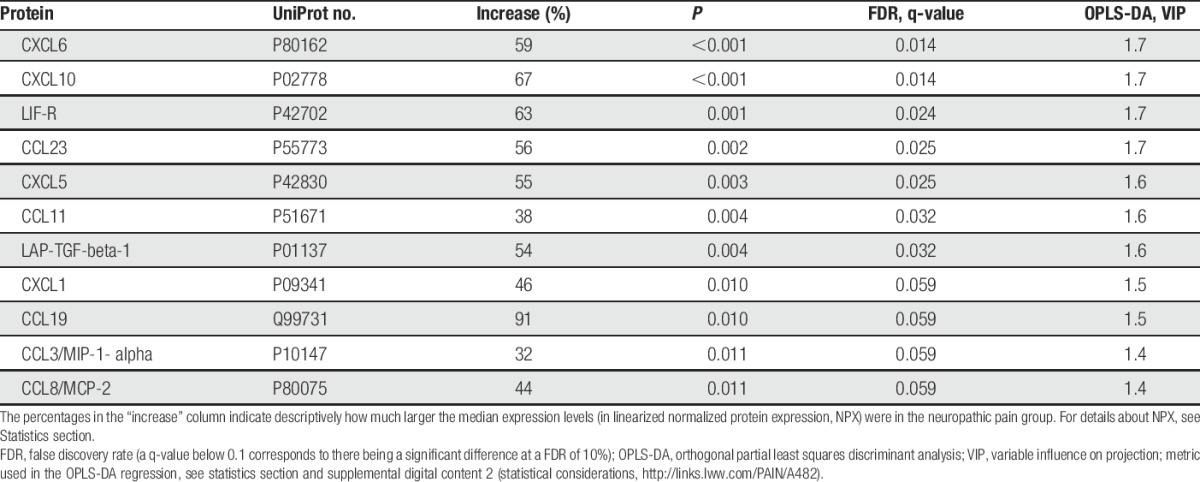
List of upregulated inflammation-related proteins in the cerebrospinal fluid of patients with neuropathic pain (cohort 1a), compared with healthy control subjects (cohort 1b), by multiple univariate testing with control of FDR and by OPLS-DA.

**Figure 2.**
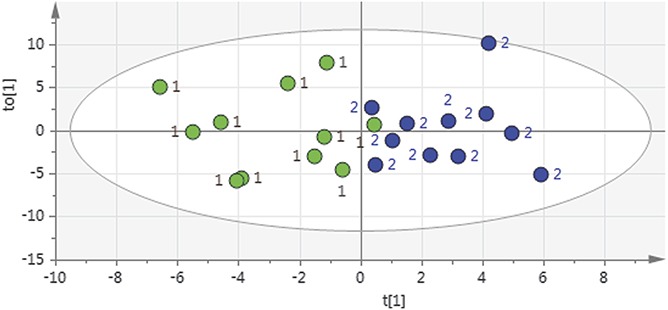
Two-dimensional score plot of orthogonal partial least squares discriminant analysis comparing inflammation-related proteins in the cerebrospinal fluid of patients with neuropathic pain (cohort 1a) with healthy control subjects (cohort 1b). Class separation between neuropathic pain patients (n = 11, green dots marked “1”) and healthy controls (n = 11, blue dots marked “2”) occurs along the t[1] axis (interclass variation). The to[1] axis represents intraclass variation. The ellipse represents the Hotelling T^2^ 95% confidence interval used when identifying strong outliers.

Cohorts 1a and 1b were overviewed with PCA (2 principal components, *R*^2^ = 0.62, *Q*^2^ = 0.46); no outlier was found. Then, an OPLS-DA model was computed (1 predictive intraclass latent variable and 1 interclass latent variable, *R*^2^ = 0.67 and *Q*^2^ = 0.43), showing clear separation between patients and healthy control subjects (*P* = 0.038 by CV-ANOVA; Fig. 2). Eleven proteins had VIP of >1.3 (ie, were very important for group discrimination), and these were the same as the ones listed above using the FDR; the VIP values of OPLS-DA are shown in Table 2.

### 3.2. Patients with neuropathic pain (cohort 2) versus healthy control subjects (cohort 1b)

The cohort 2 patients (n = 16) did not significantly differ from the cohort 1b healthy controls (n = 11) concerning age (56 [46-68] years vs 54 [44-57] years, respectively; *P* = 0.178) and sex (69% vs 64% women, respectively; *P* = 1.0). Detailed individual characteristics for cohort 2 are shown in Table 3. Data on 50 inflammation-related proteins were available.

**Table 3:**
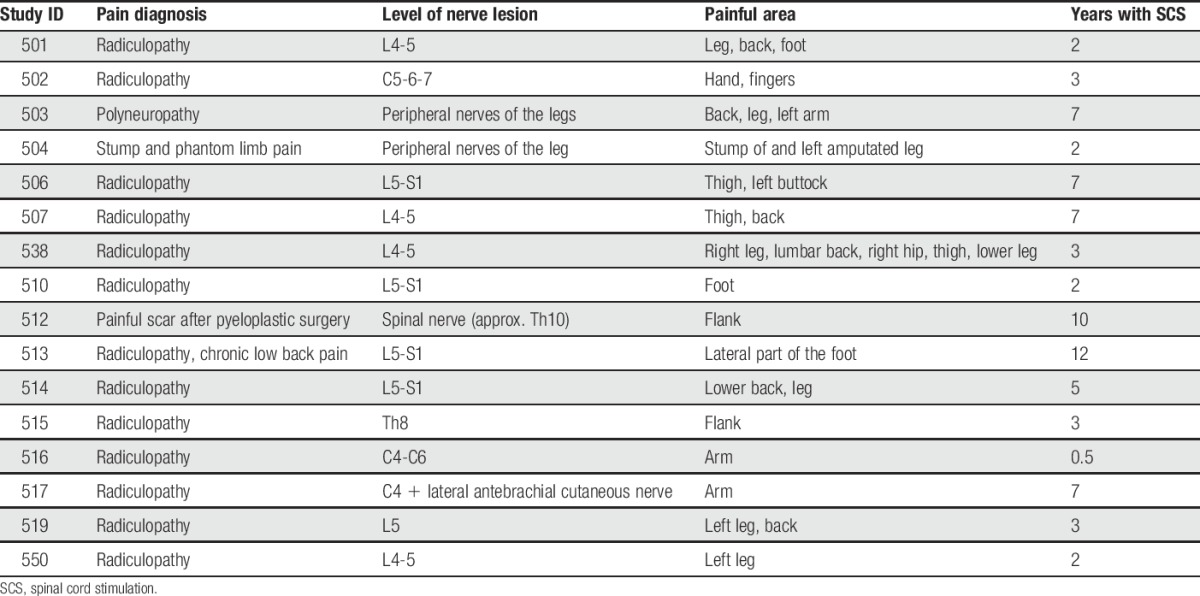
Neuropathic pain characteristics for cohort 2 (n = 16).

Cohorts 2 and 1b were overviewed with PCA (2 principal components, *R*^2^ = 0.69, *Q*^2^ = 0.57); no outlier was found. An OPLS-DA model was computed for cohort 2 versus cohort 1b (1 predictive intraclass latent variable and 2 interclass latent variables, *R*^2^ = 0.90 and *Q*^2^ = 0.66; *P* < 0.001 by CV-ANOVA). Eleven proteins had VIP of >1.3 and were upregulated in patients: LAPTGF-beta-1, CCL11, 4E-BP1, CXCL10, CCL23, CX3CL1, CXCL6, CD5, CCL8, CCL25, and CXCL11 (in falling order of VIP, range, 1.75-1.31).

### 3.3. Overlap between the 2 neuropathic pain cohorts

We found a 55% overlap when comparing the top 11 proteins of the 2 OPLS-DA models: LAPTGF-beta-1, CCL11, CXCL10, CCL23, CXCL6, and CCL8 were common to both models. LIF-R, CXCL1, CCL19, CXCL5, and CCL3 were specific for cohort 1a, whereas 4E-BP1, CX3CL1, CD5, CCL25, and CXCL11 were specific for cohort 2. Among the proteins specific for either cohort, the following 5 proteins had VIP 1.0 to 1.3 in the other cohort, that is, were somewhat (albeit not very strongly) associated with NeuP in the other cohort as well: CXCL1, CXCL5, CXCL11, and CX3CL1.

## 4. Discussion

We have determined an extensive CSF inflammatory profile of patients with severe peripheral NeuP who were candidates for (cohort 1a) or had an on-going (cohort 2) treatment with SCS, compared with healthy control subjects (cohort 1b).

### 4.1. The question of reproducibility

The same panel has recently been used for serum profiling of NeuP patients,^55^ but this is the first time that such a “holistic” CSF inflammatory fingerprint has been described for NeuP. We have also recently used the same panel on CSF from patients with fibromyalgia,^13^ with a remarkable overlap of results with the present study: all 6 proteins upregulated in both NeuP cohorts (5 of which were chemokines) were also major findings in patients with fibromyalgia. Even though it has to be acknowledged that the present study and the fibromyalgia study shared the same CSF control group, the overlap of results is nonetheless striking. Recent plasma–serum studies using the same multiplex panel^13,30,55^ have also shown remarkable overlaps in results (different cohorts of patients and different control groups). Statistical considerations are discussed in supplemental digital content 2 (available online at http://links.lww.com/PAIN/A482).

A large part of the main findings of cohort 1a could be reproduced in cohort 2, with an overlap of 55% concerning the top 11 proteins. The difference between the top 11 proteins could perhaps be due to the fact that the 2 NeuP cohorts differed concerning the presence or absence of long-term SCS treatment. Even though the 2 cohorts consisted of more or less the same category of patients, it is conceivable that long-term modulatory effects of SCS might have altered the CSF inflammatory profile of cohort 2. Even though the 2 NeuP cohorts were compared with the same control group, which is an obvious limitation, the actual overlap of results between the 2 comparisons is still noteworthy. Also, both patient cohorts were highly refractory to conventional treatment, and our results cannot be generalized to any NeuP.

Given that levels of 15 of 63 cytokines have been shown to be associated with age (albeit in plasma),^44^ the fact that the groups did not differ statistically concerning age and sex is important to underline.

### 4.2. Chemokines and neuroinflammation

A description of the chemokine family, and of LAPTGF-beta-1, can be found in supplemental digital content 3 (available online at http://links.lww.com/PAIN/A482). Strikingly, levels of LAPTGF-beta-1 have also been increased in all the other studies that we have hitherto performed with the present panel (including the present study).^13,30,55^

Chemokine receptors are potential pharmacological targets.^58^ Chemokines can induce NeuP-like behavior in mice via bidirectional neuron–glia interactions.^35^ The contribution of spinal chemokines, primarily CCL2 (MCP-1) and CX3CL1 (fractalkine) and also CXCL21, CXCL13 and other chemokines, to pain-like behavior in rodent models of NeuP has been extensively reviewed.^28,36^ Notably, CX3CL1 (fractalkine) is thought to be involved in a prominent pathway in the development of NeuP.^18,60^ For example, in the spinal cord dorsal horn, CX3CL1 produced by neurons has been shown to interact with microglial CX3CR1, triggering an increased production of proinflammatory cytokines such as tumor necrosis factor-alpha, interleukin (IL)-1b, and IL-6, causing central sensitization and increased pain-like behavior.^28,41^ Neuropathic animals have high CSF levels of CX3CL1.^20^ Another chemokine, CCL2, activates microglia and directly influences neurons; CCL2 can induce rapid central sensitization of dorsal horn neurons via ERK activation and enhances their excitatory synaptic transmission.^28^ The production of CXCL1 by spinal cord astrocytes has been shown to contribute to the maintenance of pain-like behavior in NeuP animal models,^17,81^ Moreover, CXCL1 has recently been found to be upregulated in the CSF samples of opioid-tolerant cancer patients.^45^ Moreover, there are indications that CXCL10 may also be involved in pain-like behavior maintenance in rodent models of NeuP.^16,37,68^ Neutralizing the action of chemokines, CCL2 or CX3CL1, attenuates nerve injury–induced pain-like behavior in rodents.^19,29,53,54,69,82^ Astrocytic chemokines can then modulate neuronal activity and potentiate synaptic transmission in the spinal cord excitatory pain circuitry.^28^ Additional chemokine references are listed in supplemental digital content 3 (available online at http://links.lww.com/PAIN/A482). The findings reported in this study are consistent with a role for chemokines in *human* NeuP.

Given the chemokines mentioned above, it is noteworthy that CX3CL1, CXCL1, and CXCL10 were part of the main findings of the present study:

(1) CX3CL1 was a main finding of cohort 2. It was not part of the main findings of cohort 1a, but a retrospective analysis revealed that it was actually upregulated in that cohort too (median linearized NPX 98% higher in patients; *P* < 0.001; VIP = 1.06) We have previously also shown that levels of CX3CL1 were high in the CSF of patients with fibromyalgia.^13^
(2) CXCL1 was elevated in cohort 1a. It was not part of the main findings of cohort 2, but it was actually upregulated in that cohort too (median linearized NPX 46% higher in patients; *P* = 0.009; VIP = 1.23).
(3) CXCL10 was a main finding in both cohorts.

To the best of our knowledge, among the other major findings of the study, neither CXCL6 nor CCL23 have been implicated in NeuP. In contrast, CCL11 has been investigated in at least 3 models of NeuP^43,49,67^; see also supplemental digital content 3 (available online at http://links.lww.com/PAIN/A482).

### 4.3. Cytokines, neurotrophic factors, and neuroinflammation

“Classical” cytokines (tumor necrosis factor-α, IL-6, IL-1b)^9,31,52,76^ and neurotrophic factors^15,48^ are discussed in supplemental digital content 3 (available online at http://links.lww.com/PAIN/A482).

### 4.4. Neuroinflammation and neuropathic pain

Although neuroinflammation is not easily defined,^26,57^ it is nonetheless a frequently used concept in modern pain medicine.^24,32^ Are we perhaps measuring some aspects of central neuroinflammation in humans? This would be a major step forward for pain medicine, as evidence of central neuroinflammation has hitherto been mostly gained through animal experiments.^32,76^

Neuroinflammation can be said to have 3 characteristic components with effects on pain behavior in animal models: (1) infiltration of immune cells,^22^ (2) activation of glial cells,^21,24,29,40,63,80^ and (3) production of inflammatory mediators.^34,36^ Neuroinflammation can contribute to central sensitizationand NeuP by chemokine^18^ and cytokine pathways.^41^

All in all, we think it is fair to say that we might have “visualized” central neuroinflammation, this being one possible mechanism associated with central sensitization, impaired descending pain inhibition, and the pain hypersensitivity characterizing chronic pain states.^38,39^

### 4.5. The question of causality

Granted that our results are valid (ie, that they really reflect pain-related pathophysiology and not, eg, a confounding effect of concomitant medicines or of other medical conditions such as the ones listed in Table 1), it is important to consider whether the CSF inflammatory fingerprint that we have described *directly* relates to the *pathophysiology* of NeuP (eg, central sensitization due to neuroinflammation) or if it is an inflammatory *risk factor* that was present prior to the development of NeuP (eg, a genetic susceptibility such as HLA haplotype^23,61,66,77^). A third possibility could be that the fingerprint is a *consequence* of NeuP, for example, mirroring pain-related stress, physical inactivity,^62^ depression,^75^ or bad sleep.^56^ Of course, all 3 of these categories may play a role. Disentangling the contribution of these potentially mutually interacting factors will be very difficult. For instance, levels of peripheral IL-6 are known to be influenced by regular exercise, individuals who are inactive having higher baseline levels of this particular cytokine.^62^

It is important to underline that cytokines and chemokines are probably not very specific biomarkers. It seems sensible to hypothesize that, in the future, biomarkers for different chronic pain conditions may fall into 2 categories, namely, on the one hand those that are common to several or perhaps even all chronic pain conditions and, on the other hand, those that are specific for a given condition. In future CSF studies, it will be important to determine unique and common markers for different pain conditions. Such studies will also have to take comorbid conditions, like depression, anxiety, and poor sleep, into consideration, as these might also be associated with chronic inflammation.^75^ That this was not done is a limitation of the study, as is the fact that we did not register factors like the level of physical activity and smoking or alcohol use, and that we did not take putative diurnal variations into consideration when planning the study.

## 5. Conclusions

Using a panel of inflammation-related proteins, we have found evidence of on-going neuroinflammation in patients with NeuP. The results from 2 cohorts of fairly comparable patients were quite similar (although not perfectly identical), showing that mainly a number of chemokines were upregulated in CSF from patients compared with healthy control subjects. We find it conceivable that we might have mirrored central neuroinflammation in this very debilitating chronic pain condition. However, further studies are needed to confirm these findings, and the question of causality remains difficult to answer. Because it has been suggested that prevalent comorbidities to chronic pain also are associated with neuroinflammation, it will be important to determine unique and common mediators.

## Conflict of interest statement

The authors have no conflict of interest to declare.

The study was supported by Uppsala Berzelii Technology Centre for Neurodiagnostics, with financing from the Swedish Governmental Agency for Innovation Systems (Vinnova) and the Swedish Research Council (grant no. P29797-1). The study was also financially supported by the Swedish Research Council (grant no. K2015-99x-21874-05-4), the County Council of Östergötland (LIO-35923, SC-2013-00395-36), and AFA Insurance (140341). The funders had no role in the study design, data collection and analysis, decision to publish, or preparation of the manuscript.

## Appendix A. Supplemental digital content

Supplemental digital content associated with this article can be found online at http://links.lww.com/PAIN/A482.
